# Circular RNA circRANGAP1/miR-512-5p/SOD2 Axis Regulates Cell Proliferation and Migration in Non-small Cell Lung Cancer (NSCLC)

**DOI:** 10.1007/s12033-023-00962-1

**Published:** 2023-12-11

**Authors:** Chunhua Zhao, Zhongqi Zhang, Zhengzuo Wang, XinLi Liu

**Affiliations:** 1 Department of Internal Medicine Oncology, Traditional Chinese Medicine Hospital of Jiashan, 38 Gujiadai, Jiaxing, 314100 Zhengjiang China; 2 Department of Proctology, Traditional Chinese Medicine Hospital of Jiashan, 38 Gujiadai, Jiaxing, 314100 Zhengjiang China; 3 Department of Digestive Oncology, Cancer Hospital of China Medical University, Liaoning Cancer Hospital & Institute, No. 44 Xiaoheyan Road, Shenyang, 110042 Liaoning China

**Keywords:** NSCLC, circRANGAP1, miR-512-5p, SOD2, ceRNA

## Abstract

Non-small cell lung cancer (NSCLC) is the most prevalent histology type of lung cancer worldwide, accounting for 18% of total cancer-related deaths estimated by GLOBOCAN in 2020. CircRNAs have emerged as potent regulators of NSCLC development. CircRANGAP1 (hsa_circ_0001235/hsa_circ_0063526) is a potential biomarker for NSCLC identified by microarray dataset analysis. Here, we investigated the biological functions of circRANGAP1 in NSCLC development and elucidated the associated competing endogenous RNA (ceRNA) mechanisms. We found that circRANGAP1 expression was upregulated in NSCLC tissues and cells, which was inversely correlated with carcinogenesis and poor clinical outcome of NSCLC patients. CircRANGAP1 knockdown inhibited NSCLC migration by regulating miR-512-5p/SOD2 axis. In conclusion, circRANGAP1 facilitated NSCLC tumorigenesis and development by sponging miR-512-5p to upregulate SOD2 expression. Suppression of circRANGAP1 expression by si-circRANGAP1 treatment could be a strategy to inhibit NSCLC development and metastasis.

## Introduction

Lung cancer accounts for 18% of total cancer-related deaths as per GLOBOCAN 2020 [1]. Non-small cell lung cancer (NSCLC) is the most prevalent subtype of lung cancer [2]. Despite advances in NSCLC treatment, the overall cure and survival rate of patients remain low [3]. It is therefore necessary to explore and understand the molecular mechanism of NSCLC development and progression to improve the survival of patients with NSCLC.

Circular RNAs (circRNAs) represent a novel cluster of single-stranded non-coding RNAs and are characterized by a closed loop structure with a canonical splicing junction site [4]. Noncoding RNAs including long non-coding RNAs (lncRNA), miRNAs, and more recently characterized circRNA have been found to regulate various biological processes during healthy or disease states [4]. Reportedly, circRNAs, especially NSCLC, are involved in the modulation of various diseases [5], such as diabetic cardiomyopathy [6], osteoarthritis [7], and cancers [8, 9]. For example, downregulation of circ-YES1 suppresses NSCLC migration and proliferation through the miR-142-3p-HMGB1 axis [6]. CircRNA OXCT1 promotes the malignant progression and glutamine metabolism of NSCLC by absorbing miR-516b-5p and upregulating SLC1A5 [10]. Of note, circRANGAP1 has been found to be upregulated in multiple cancers and affected different oncogenic processes [11, 12]. The latest research showed that circRANGAP1 plays an important role in NSCLC [13]. However, the molecular mechanism by which circRANGAP1 regulates NSCLC progression is largely unknown.

In the current study, we aimed to investigate the biological functions and the regulatory mechanisms of circRANGAP1 in NSCLC tumorigenesis and development, thus providing a new biomarker that would facilitate the development of therapeutic strategies against NSCLC in clinical practices.

## Materials and Methods

### Reagents

Dulbecco’s modified Eagle medium (DMEM) and fetal bovine serum (FBS) were purchased from Solarbio Inc. (Beijing, China). CCK8 assay kit was obtained from Dojindo Corp (Kyushu, Japan). RNase® was provided by Sigma-Aldrich Inc. (St. Louis, USA). Bestar qPCR RT kit and Bestar qPCR MasterMix kit were obtained from DBI Bioscience Inc. (Beijing, China). Lipofectamine 3000 was obtained from Invitrogen Inc. (Carlsbad, USA). RIPA Cell Lysis Buffer and PMSF were purchased from Beyotime Biotechnology (Nanjing, China). PVDF membrane was provided by Millipore Inc. (Boston, USA). Pierce™ BCA Protein Assay Kit was provided by Thermo Scientific Inc. (Waltham, USA). Antibodies against SOD2 and GAPDH were purchased from Abcam Inc. (Boston, USA). The pGL3 luciferase reporter vector and Dual-Luciferase Reporter Assay System were bought from Promega Inc. (Shanghai, China).

### Patients and Informed Consents

Cancerous tissues and adjacent normal tissues were collected from 60 NSCLC patients with informed consent during surgical treatment at XXX Hospital. Tissue specimens were frozen immediately in liquid nitrogen and kept at -80 °C until use. All experimental protocols were approved by the Ethics Committee of XXX Hospital. The study was performed following the Declaration of Helsinki revised in 2013.

### Microarray Data

Significantly, differentially expressed circRNAs (DEcircRNAs) were identified by screening the GSE158695 dataset of the international public database of Gene Expression Omnibus (GEO, http://www.ncbi.nlm.nih.gov/geo) using the online tool GEO2R with a cut-off criterion of |logFC (foldchange)|> 1, *p* < 0.05.

### Target Gene Prediction

The putative miRNAs sponged by circRANGAP1 were predicted with the circular RNA interactome (https://circinteractome.nia.nih.gov/). The target mRNAs of miR-512-5p were predicted with TargetScan (http://www.targetscan.org/).

### Cell Lines and Cell Cultures

The human NSCLC cell lines (H1229, A549, SPCA1, and CALU3) and the human bronchial epithelial cell line (16HBE) were all provided by the Cell Bank of Chinese Academy of Sciences (Shanghai, China). Cells were maintained in Dulbecco’s Modified Eagle’s medium with 10% fetal bovine serum at 37 °C in a 5% CO_2_ atmosphere.

### Dual-Luciferase Reporter Assays

A pGL3 luciferase reporter vector containing miR-1184 mimic or negative control (NC) and the circRANGAP1-WT/-MUT or SOD2-WT/-mutation sequence were constructed and co-transfected into cells (1 × 10^4^ cells/well) seeded in a 24-well cell culture plate by Lipofectamine 3000. After culturing for 24 h, the luciferase activity was detected with a Dual-Luciferase Reporter Assay System and was normalized to the internal control (Renilla luciferase).

### RNA Stability Examination

The RNA stabilities of the linear CSNK1G1 and the circRANGAP1 were investigated by treatment using 5-U/μg RNase® for 20 min at 37 °C. Then, RNA was purified and quantified with qRT-PCR.

### Quantitative Real-Time PCR (qRT-PCR) Assay

Total RNA was purified from NSCLC cells and tissues using TRIzol, and the concentration was determined with a NanoDrop (Thermo Fisher Scientific, USA). cDNA was inversely transcribed using a Bestar qPCR RT kit. qRT-PCR assay was performed on a 7500 Fast Real-Time PCR system (Applied Biosystems, USA) using a Bestar qPCR MasterMix kit following the manufacturer’s guidelines. Relative gene expressions were calculated with the 2^−ΔΔCt^ method. U6 (for miRNA quantification) and GAPDH (for quantification of circRNAs and mRNAs) were used as internal controls.

### Western Blot

Cells were washed twice with pre-cold PBS. Total proteins were extracted from cells with RIPA buffer containing 10% PMSF and were quantified with the Pierce™ BCA Protein Assay Kit. The proteins were resolved by 10% SDS-PAGE and transferred onto a PVDF membrane. The membrane was blocked at room temperature in 5% non-fat milk for 1 h, followed by incubation with diluted primary antibody overnight kept at 4 °C. Next, the membrane was treated with secondary antibodies conjugated to horseradish peroxidase at RT for 1 h. The blot was developed with chemiluminescent substrates and visualized by ChemiDoc Touch Imaging System (Bio-Rad, Hercules, USA).

### Cell Migration Assay

Transwell assay was implemented to detect cell migration ability (Corning Costar, Tewksbury, USA). Briefly, 500 μL of medium containing 10% FBS was applied in the lower chamber and 300 μl of serum-free medium (2 × 10^5^ cells) was loaded in the upper chamber of a 24-well Transwell. After 24 h of incubation, migrated cells were fixed in 4% paraformaldehyde, stained in crystal violet, and then counted under a microscope.

### CCK8 Assay

Cell viability was measured with the CCK8 assay according to the manufacturer’s instructions. Briefly, 10 μL of CCK-8 reagent was added into 100 μL of cell culture in a 96-well plate. The optional density (OD) value was read at 450 nm after incubation for 4 h at 37 °C.

### Colony Formation Assay

NSCLC cells were seeded in a 6-well plate with an initial concentration of 3000 cells/well and were incubated under 5% CO_2_ in a cell incubator for two weeks. Cell colonies were then imaged and counted under a microscope after fixing in 4% paraformaldehyde and staining with crystal violet.

### Xenograft Animal Model

Eighteen 4-week-old SCID mice were provided by Shanghai SLAC Laboratory Animal Co, Ltd. (Shanghai, China) and were maintained under pathogen-free conditions in the Laboratory Animal Center of XXX University. All experiments were conducted following the ethical guidelines for animal experiments. About 1 × 10^5^ A549 cells transfected with si-circRANGAP1 or scramble (si-NC) in 100 μL of serum-free medium were subcutaneously injected into the right flanks of each mouse. Tumor volume was routinely measured once a week. Tumors were dissected and weighed after the mice were sacrificed by anesthesia and cervical dislocation.

### Statistical Analyses

Statistical analyses were carried out using the GraphPad Prism (La Jolla, USA). Data were presented as mean ± standard deviation. Two-tailed Student’s *t* test was used for two-group comparison, and One-way ANOVA for the comparison among three or multiple groups. Pearson correlation analysis was performed to assess gene association. Kaplan–Meier curves were plotted to examine survival differences between patient groups. *P* < 0.05 was considered statistically significant.

## Results

### Increased circRANGAP1 Expression Correlates with Poor Clinical Outcome in NSCLC

**Fig. 1:**
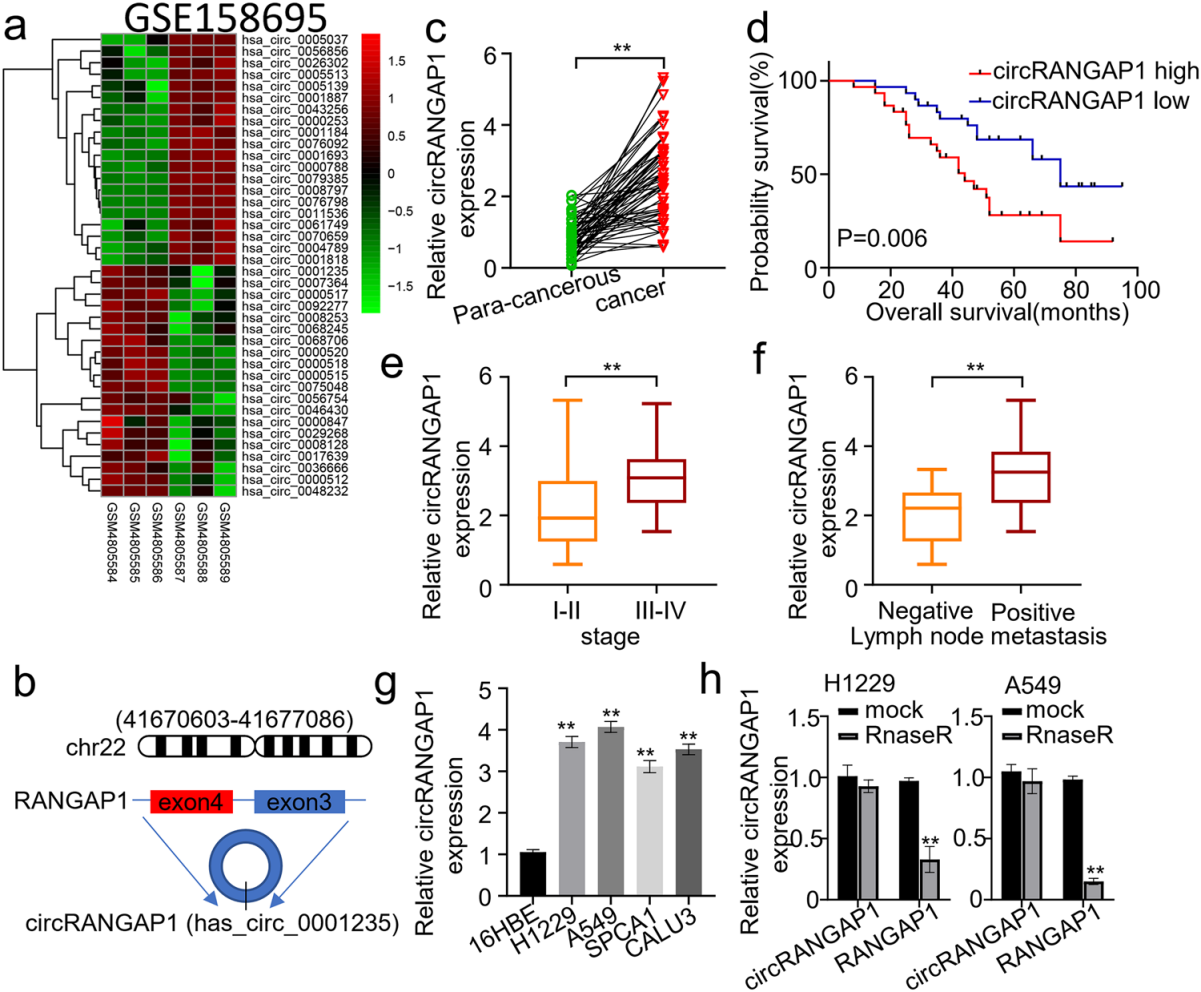
circRANGAP1 (has_circ_0001235) is upregulated in NSCLC and associated with poor outcome of NSCLC patients. Differently expressed circRNAs (DEcircRNAs) in human NSCLC and the corresponding non-cancerous tissues were compared using the online GEO2R tool based on the human circRNA microarray data of GSE158695 (*n* = 3) from GEO database (|logFC|> 1, *P* < 0.05). **a** Heatmap of the 20 most significant DEcircRNAs including circRANGAP1 (has_circ_0001235) in NSCLC tissues. **b** circRANGAP1 is formed by reverse splicing of the exons 3 and 4 of the host gene RANGAP1. **c** qRT-PCR determined circRANGAP1 expression levels in the cancerous and the adjacent normal tissues of NSCLC patients (*n* = 60). **d** qRT-PCR determined circRANGAP1 expression levels in the NSCLC (H1229, A549, SPCA1, and CALU3) cells and the human bronchial epithelial (16HBE) cells. **e** Using the median expression level of circRANGAP1 as the cut-off value, the overall survival of the 60 NSCLC patients with high versus low circRANGAP1 expression levels was plotted with Kaplan–Meier curves. **f** Association between the circRANGAP1 expression level and the stages of the 60 NSCLC patients. **g** Association between the circRANGAP1 expression level and the lymph node metastasis status of the 60 NSCLC patients. **h** Resistance of circRANGAP1 to ribonuclease R (Rnase R) in H1229 and A549 cells. **p < 0.01

To explore the potential circRNAs that are involved in NSCLC development, bioinformatics data of gene expressions in NSCLC were obtained from the GSE158695 dataset in the GEO database (*n* = 3). The 20 most significant differentially expressed circRNAs were visualized by a Heatmap. Among them, circRANGAP1 was identified to be a significantly upregulated one (Fig. 1a). We next characterized circRANGAP1 as a novel circRNA by examining its physical circular structure. CircRANGAP1 was formed by the reverse splicing of the exon 3 and 4 of the host gene *RANGAP1* (Fig. 1b). To further validate circRANGAP1 expression levels in NSCLC, qRT-PCR assay was performed. CircRANGAP1 expression was significantly upregulated in NSCLC tissues and NSCLC cells (H1229, A549, SPCA1, and CALU3) compared with the adjacent normal tissues and 16HBE cells (Fig. 1c and g). To assess whether circRANGAP1 expression was associated with clinical prognosis in NSCLC patients, the 60 NSCLC patients were divided into two groups using the median circRANGAP1 expression level as a cut-off value. The group with higher circRANGAP1 expression level showed a significantly shorter overall survival time (Fig. 1d). Furthermore, we correlated circRANGAP1 expression level with clinical characteristics, and we found that circRANGAP1 expression was significantly upregulated in NSCLC patients at advanced clinical stages (III-IV) and with positive lymph node metastasis (Fig. 1e and f). Moreover, to detect the stability of circRANGAP1, H1229 and A549 cells were treated with RNase R (RNA synthesis inhibitor). QRT-PCR results showed that circRANGAP1 was more capable of resistance to RNase R digestion (Fig. 1h).

Together, these findings suggested the stability of circRANGAP1 and upregulated circRANGAP1 is associated with the poor clinical outcome of NSCLC patients.

### Knockdown of circRANGAP1 Suppresses NSCLC Cell Proliferation and Migration In Vitro and Tumor Growth In Vivo

**Fig. 2:**
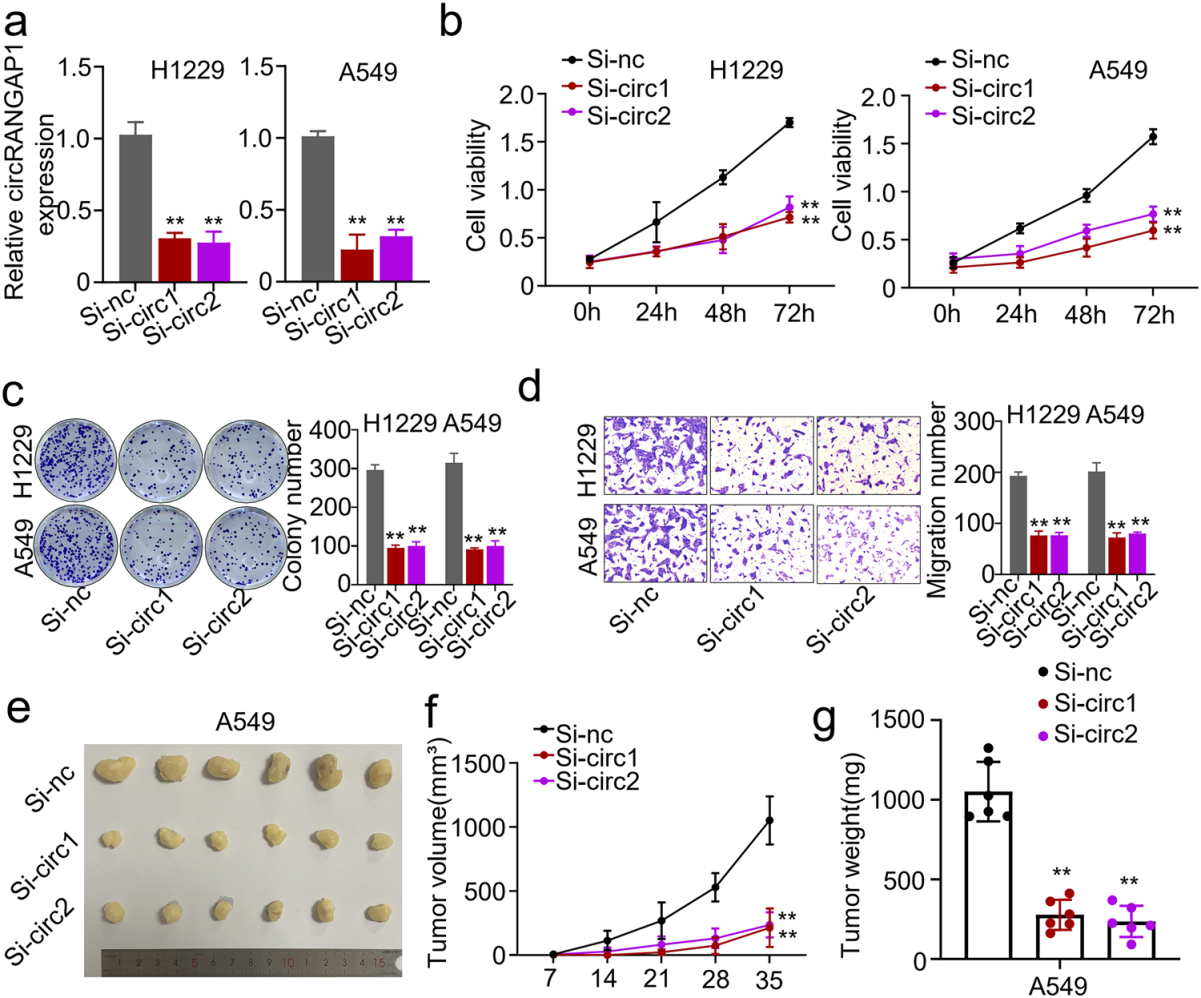
circRANGAP1 silence suppresses the proliferation and metastasis of non-small cell lung cancer cells in vivo and in vitro. **a** qRT-PCR analysis confirmed that circRANGAP1 was successfully silenced with si-circRANGAP1 in the H1229 and A549 cells. **b**–**c** CCK8 (**b**) and colony formation (**c**) were used to analyze proliferation of H1229 and A549 cells after circRANGAP1 silence. **d** Transwell assay was used to analyze the migration ability of H1229 and A549 cells after circRANGAP1 silence. **e** Tumor mass, **f** tumor volume, and **g** tumor weight in xenograft mouse model prepared with A549 cells with or without circRANGAP1 silence. **p < 0.01

**Fig. 3:**
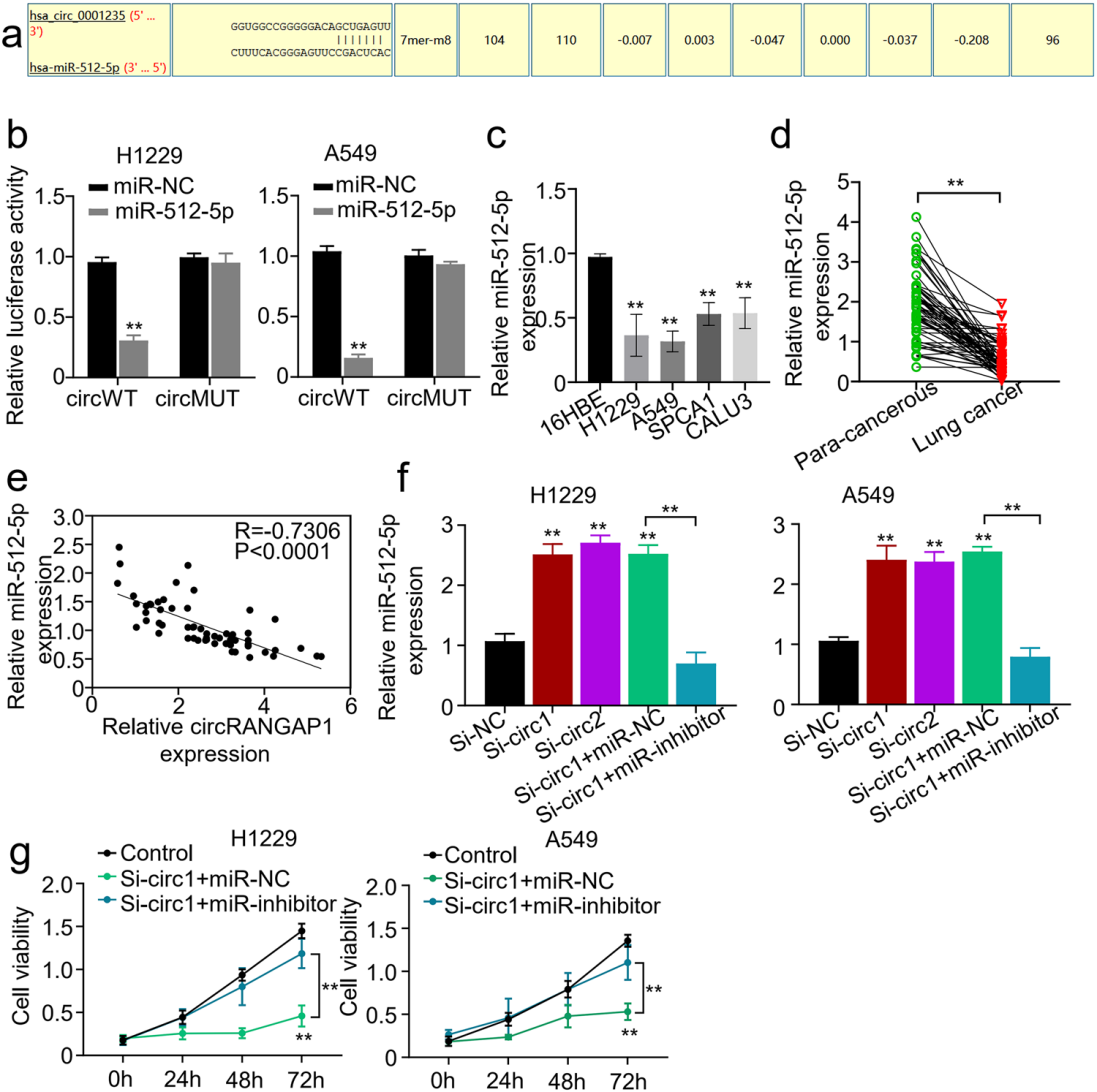
circRANGAP1 serves as a sponge for miR-512-5p. **a** Schematic representation of the potential binding sites of circRANGAP1 with miR-512-5p predicted with the online circular RNA interactome tool (https://circinteractome.nia.nih.gov/). **b** Dual-luciferase reporter gene activity assay in the H1229 and A549 cells co-transfected with circRANGAP1 wild-type (circWT) or mutated reporter (circMUT) and miR-512-5p mimics (miR-512-5p). **c** qRT-PCR determined miR-512-5p expression levels in the NSCLC (H1229, A549, SPCA1, and CALU3) cells and the human bronchial epithelial (16HBE) cells. **d** qRT-PCR determined miR-512-5p expression levels in the paired cancerous and adjacent normal tissues from 60 NSCLC patients. **e** Pearson correlation analyzed association between miR-512-5p with circRANGAP1 in the cancerous tissues from 60 NSCLC patients. **f** qRT-PCR determined miR-512-5p expression levels in the H1229 and A549 cells after circRANGAP1 silence with or without co-transfection of miR-512-5P inhibitor. **g** CCK8 analyzed viabilities of H1229 and A549 cells after circRANGAP1 silence with or without co-transfection of miR-512-5P inhibitor. **p < 0.01

To confirm the function of circRANGAP1 in NSCLC in vitro, loss-of-function analyses were performed with circRANGAP1-specific si-RNA (Fig. 2a). QPCR confirmed that the high knockdown efficiency of circRANGAP1 si-RNA in H1229 and A549 cells (Fig. 2a). The cell viability of H1229 and A549 cells was determined by CCK-8 assay. The results showed that circRANGAP1 knockdown significantly decreased the cell viability (Fig. 2b). Meanwhile, colony formation assay showed that circRANGAP1 knockdown significantly decreased the colony formation ability of H1229 and A549 cells (Fig. 2c). Furthermore, transwell assay showed that circRANGAP1 knockdown significantly decreased the migration ability of H1229 and A549 cells (Fig. 2d). These results indicated that knockdown of circRANGAP1 suppresses the proliferation and migration of NSCLC cells in vitro. Subsequently, we established a xenograft tumor model in nude mice to investigate the effect of circRANGAP1 on NSCLC growth in vivo. The results showed that circRANGAP1 knockdown negatively affected tumor volume (Fig. 3e and f) and tumor weight (Fig. 3g). In summary, our data demonstrated the functional importance of circRANGAP1 in promoting NSCLC tumor growth in vivo*.*

### CircRANGAP1 Functions in NSCLC Through Sponging miR-512-5p

We next investigated the detailed mechanism of circRANGAP1-mediated NSCLC progression. CircRANGAP1 frequently function as a competing endogenous RNA (ceRNA) to sponge miRNAs and then regulate gene expression. We predicted the potential miRNAs targeted by circRANGAP1 using Circular RNA Interactome (https://circinteractome.nia.nih.gov/) and found that miR-512-5p was a target of circRANGAP1 (Fig. 3a). Furthermore, to prove the circRANGAP1–miRNA interaction, dual-luciferase reporter assay was performed and showed a decreased luciferase activity in H1229 and A549 cells that were transfected with wild-type circRANGAP1 and miR-512-5p mimics (Fig. 3b). Moreover, qRT-PCR revealed that miR-512-5p was significantly downregulated in the NSCLC cells (Fig. 3c) and cancerous tissues (Fig. 3d). The association between circRANGAP1 and miR-512-5p was analyzed by the Pearson correlation coefficient. This analysis showed that circRANGAP1 expression was negatively associated with miR-512-5p expression in the cancerous tissues of the 60 NSCLC patients (Fig. 3e). Meanwhile, we next studied the functional roles of circRANGAP1–miR-512-5p interaction in NSCLC, miR-512-5p expression was significantly upregulated after circRANGAP1 knockdown in H1229 and A549 cells, but was not in the presence of miR-512-5p inhibitor (Fig. 3f). CCK8 assay revealed that the viability of H1229 and A549 cells was significantly inhibited in the circRANGAP1 knockdown group, which was almost restored by co-transfection of miR-512-5p inhibitor (Fig. 3g). These findings together suggested that circRANGAP1 functioned as a sponge of miR-512-5p in NSCLC cells.

### SOD2 is a Target Gene of miR-512-5p

**Fig. 4:**
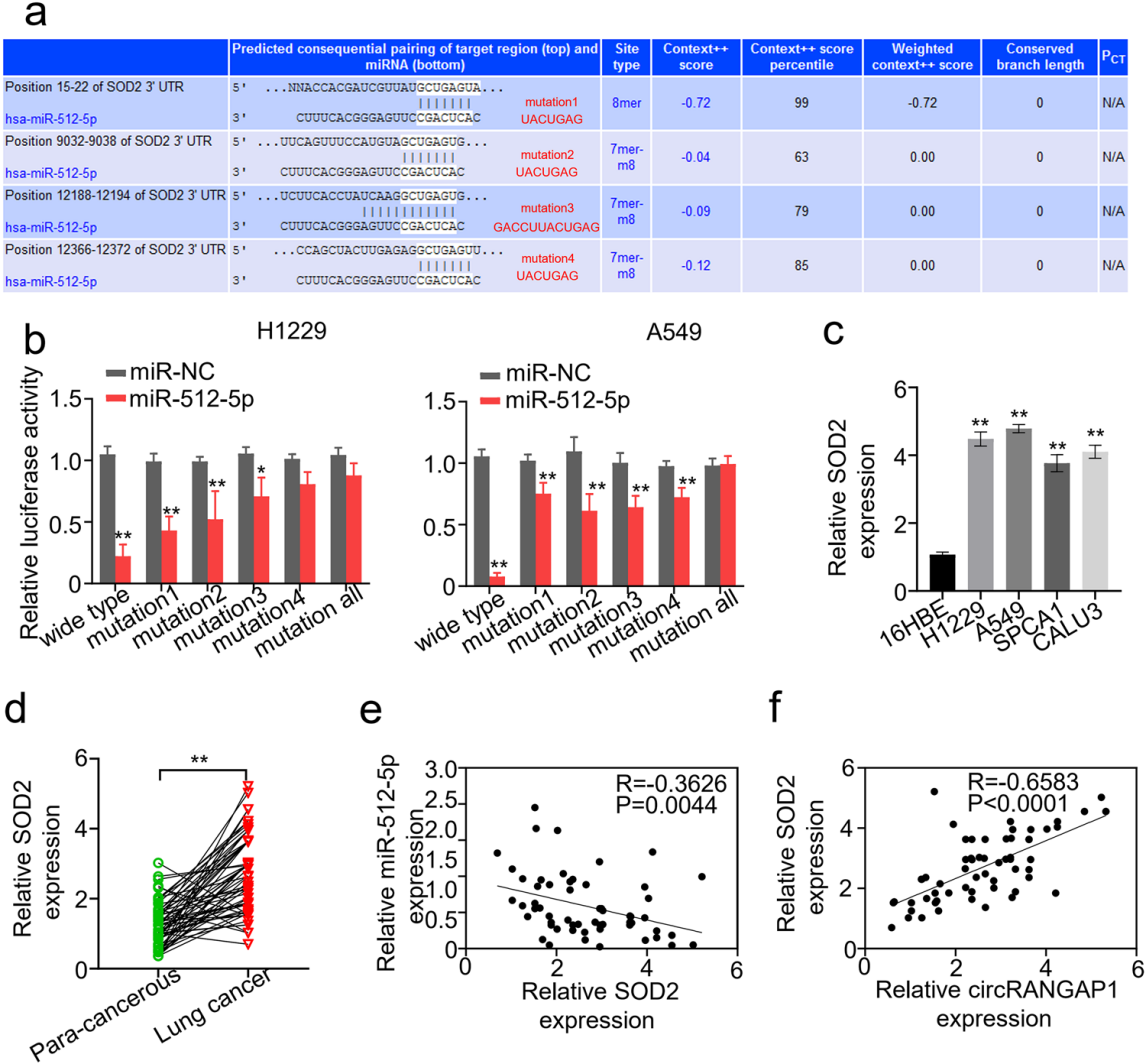
SOD2 is the target gene of miR-512-5P. **a** Schematic representation of the potential binding sites of miR-512-5p with the SOD2 3' UTR predicted by the online tool TargetScan (http://www.targetscan.org/). **b** Dual-luciferase reporter gene activity assay in the H1229 and A549 cells co-transfected with SOD2 wild-type or different mutated reporter (mutation) and miR-512-5p mimics (miR-512-5p). **c** qRT-PCR determined SOD2 expression levels in the NSCLC (H1229, A549, SPCA1, and CALU3) cells and the human bronchial epithelial (16HBE) cells. **d** qRT-PCR determined SOD2 expression levels in the paired cancerous and adjacent normal tissues from 60 NSCLC patients. **e** Pearson correlation analyzed association between SOD2 with miR-512-5p and circRANGAP1 in the cancerous tissues from 60 NSCLC patients. **f** Pearson correlation analyzed association between SOD2 and circRANGAP1 in the cancerous tissues from 60 NSCLC patients. **p < 0.01

To verify that miR-512-5p suppresses tumor progression, we used bioinformatic methods to identify the target genes of miR-194-5p. Targetscan (http://www.targetscan.org/) revealed that miR-512-5p contained potential binding sites with the 3’ UTR of *SOD2* (Fig. 4a). The binding of miR-512-5p to SOD2 was further verified. Dual-luciferase report assay displayed that miR-512-5p overexpression combined with the mimics inhibited the luciferase activity of SOD2 wild-type in both H1229 and A549 cells, while the luciferase activity of SOD2 mutant did not change significantly (Fig. 4b), hinting at a direct interaction between miR-512-5p and the 3’UTR of *SOD2*. Moreover, SOD2 expression was determined in the NSCLC cells and tissues. SOD2 expression was significantly upregulated in NSCLC cells (Fig. 4c) and in the cancerous tissues (Fig. 4d). In addition, SOD2 expression in the NSCLC tissues of 60 NSCLC patients was negatively related to miR-512-5p expression (Fig. 4e), while positively related to circRANGAP1 expression (Fig. 4f).

### circRANGAP1 Knockdown Inhibits NSCLC Cell Proliferation and Migration by Regulating miR-512-5p/SOD2 Axis

**Fig. 5:**
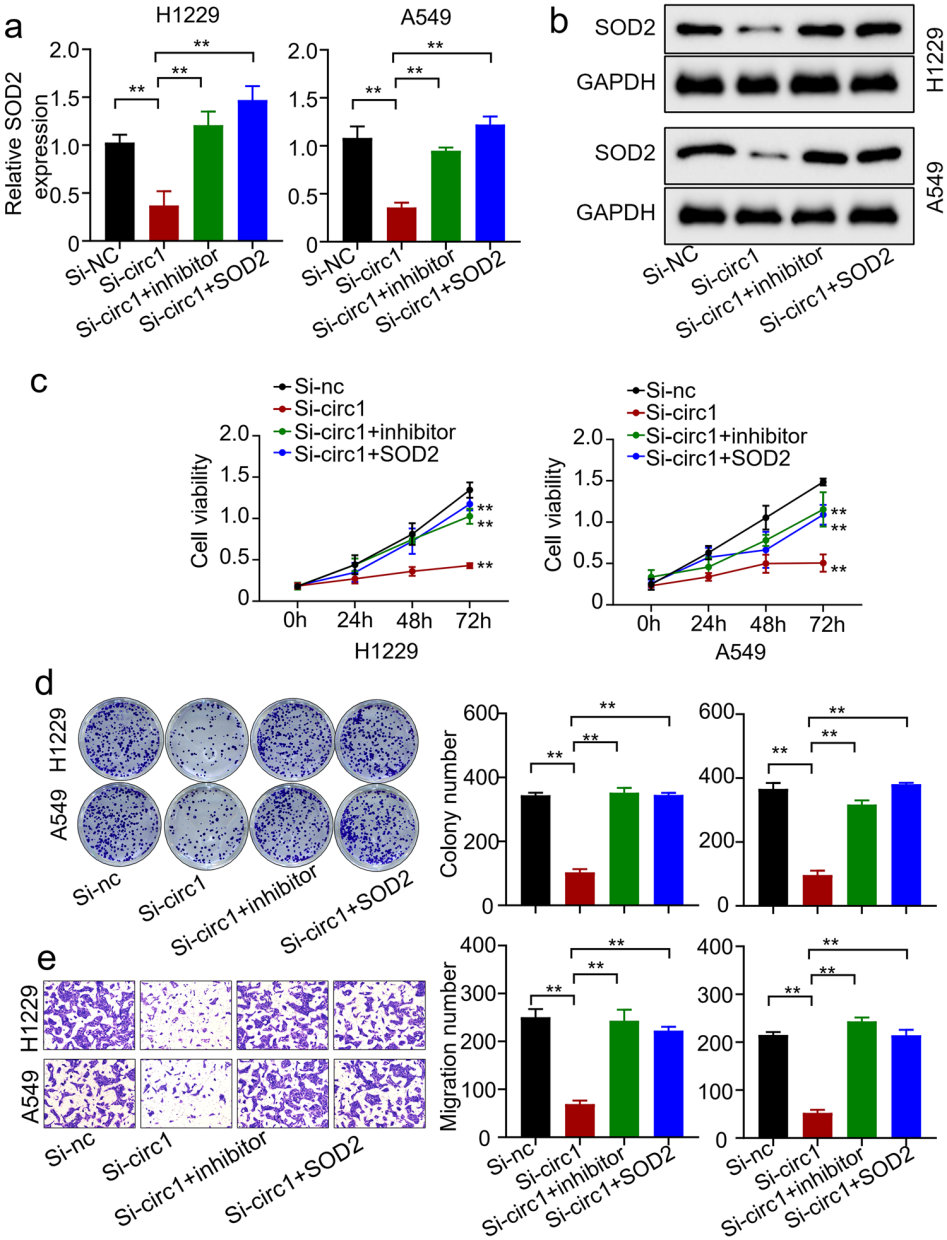
CircRANGAP1 promotes non-small cell lung cancer development by regulating miR-512-5p/SOD2 axis. **a** qRT-PCR determined SOD2 expression levels in H1229 and A549 cells after circRANGAP1 silence with or without co-transfection of miR-512-5P inhibitor or SOD2 overexpression plasmid. **b** Western blot determined SOD2 expression levels in H1229 and A549 cells after circRANGAP1 silence with or without co-transfection of miR-512-5P inhibitor or SOD2 overexpression plasmid. **c**–**d** CCK8 (**c**) and colony formation (**d**) were used to analyze proliferation of H1229 and A549 cells after circRANGAP1 silence with or without co-transfection of miR-512-5P inhibitor or SOD2 overexpression plasmid. **e** Transwell assay was used to analyze the migration ability of H1229 and A549 cells after circRANGAP1 silence with or without co-transfection of miR-512-5P inhibitor or SOD2 overexpression plasmid. **p < 0.01

Given that SOD2 is the downstream target of miR-512-5p and circRANGAP1, we further evaluated whether circRANGAP1 promoted PDAC progression through miR-512-5p/SOD2 axis; we first investigated SOD2 expression levels by qRT-PCR and western blot in NSCLC cells after circRANGAP1 knockdown with or without co-transfection of miR-512-5p inhibitor or SOD2 overexpression plasmid. The results showed that the mRNA and protein levels of SOD2 were downregulated when circRANGAP1 expression was knocked down, which were restored by co-transfection of either miR-512-5p inhibitor or SOD2 overexpression plasmid in H1229 and A549 cells (Fig. 5a and b). Moreover, functional experiments showed that circRANGAP1 knockdown inhibited cell proliferation (Fig. 5c and d) as well as migration abilities (Fig. 5e) in H1229 and A549 cells, and these defects were partially rescued by co-transfection of miR-512-5p inhibitor or SOD2 overexpression. These findings indicate that circRANGAP1 knockdown inhibits the proliferation and migration of NSCLC cells by regulating the miR-512-5p/SOD2 axis.

## Discussion

CircRNAs have been reported as multifaceted regulators of the oncogenesis of multiple cancer types, including NSCLC [14, 15]. This indicates that circRNAs can be developed as novel molecular biomarkers for early diagnosis and therapeutic targets for treatment.

CircRANGAP1 affects various physiological processes to modulate cancer progression. For example, circRANGAP1 regulates VEGFA expression by targeting miR-877-3p to facilitate gastric cancer invasion and metastasis [11]. The circRanGAP1/miR-27b-3p/NRAS axis can promote the progression of hepatocellular carcinoma [11]. In the current study, using the GEO2R online tool, we found that circRANGAP1 is a novel NSCLC-associated circRNA in the GSE dataset. QRT-PCR confirmed that circRANGAP1 was upregulated in both NSCLC tissues and cells. Furthermore, functional assays revealed that downregulation of circRANGAP1 inhibited NSCLC cell proliferation and migration. Moreover, xenograft tumor analysis confirmed that circRANGAP1 knockdown inhibited NSCLC cell growth in vivo. These findings suggest that circRANGAP1 acts as an oncogenetic factor in NSCLC development and metastasis, thus providing a potential biomarker for early diagnosis and a therapeutic target for NSCLC patients. Our findings are largely consistent with the conclusion of Chen et al. [13]. Nevertheless, our work elucidated a novel and independent regulatory signaling, which corroborates the regulatory role of circRANGAP1 in NSCLC. CircRNAs sponge miRNAs to reduce the miRNA-mediated inhibition of target mRNAs, thereby regulating cellular processes in NSCLC cells to exert [15–17]. For instance, circHMGB2 drives immunosuppression and anti-PD-1 resistance in NSCLC and squamous cell carcinomas via the miR-181a-5p/CARM1 axis [18]. Circ_0070659 predicts poor prognosis and promotes NSCLC progression via the miR-377/RAB3C pathway [19]. Here, we discovered that miR-512-5p is the target of circRANGAP1. To date, miR-512-5p has been implicated in the tumorigenesis of various human malignancies, including NSCLC [20, 21]. For example, Li et al. found that circ_0010235 regulates FAM83F expression through sponging miR-512-5p, thus inhibiting cancer-acquired paclitaxel resistance in NSCLC [22]. Bin Cao et al. reported that miR-512-5p inhibits the proliferation, migration, and invasion of NSCLC cells by targeting ETS1 [23]. In our study, miR-512-5p was identified as a potential target miRNA of circRANGAP1, which was validated by the dual-luciferase reporter assay in H1229 and A549 cells and the Pearson correlation analysis in the cancerous tissues of 60 NSCLC patients. As one of the three human SOD enzymes, SOD2 converts superoxide into less harmful products, which can then be cleared from the mitochondria, to maintain the normal mitochondrial function [24]. Serum SOD2 has been shown to be positively associated with all-cause mortality in lung cancer patients [25] and resistance to EGFR-tyrosine kinase inhibitors in NSCLC [26]. This potentiates SOD2 to be a reliable cancer biomarker and a promising anti-cancer target in NSCLC. In the present study, SOD2 was predicted to be the potential target mRNA of miR-512-5p, which was validated by the dual-luciferase reporter gene activity assay in the NSCLC cells and the Pearson correlation analysis in the cancerous tissues of 60 NSCLC patients.

Previous data suggested that circRANGAP1 [12] and SOD2 [27] functions as oncogenes, while miR-512-5p acts as a tumor suppressor [28, 29]. However, whether these three genes interact with each other to regulate NSCLC progression is unknown. Our data validated the interaction of circRANGAP1/miR-512-5p/SOD2 and implicated them in regulating NSCLC progression. However, our current study used a relatively small sample size, and further studies with a larger cohort of patients are needed.

In conclusion, our study delineated a new regulator axis involving circRANGAP1/miR-512-5p/SOD2 in NSCLC development. Our findings made significant inroads into the molecular mechanism underlying NSCLC tumorigenesis and provided potential diagnostic or therapeutic biomarkers for NSCLC.

## Data Availability

The data underlying this article are available in the article. If needed, please contact the corresponding author.
